# Speculative sewing: Researching, reconstructing, and re-imagining wearable technoscience

**DOI:** 10.1177/03063127221119213

**Published:** 2022-08-16

**Authors:** Kat Jungnickel

**Affiliations:** Goldsmiths’ College, London, UK

**Keywords:** clothing, inventions, making and doing, reconstructions, sewing, citizenship

## Abstract

This article contributes to Science and Technology Studies (STS) literatures on ‘making and doing’ by describing and analysing the practice of researching, reconstructing, and reimagining archival clothing patent data. It combines feminist speculation and reconstruction practices into what I term ‘speculative sewing’. This involves stitching data, theory and fabric into inventions described in patents and analysing them as three-dimensional arguments. In the case here, of 1890s British women’s convertible cycle wear, I examine how inventors used new forms of clothing to challenge socio-political restrictions on women’s bodies in public space and help them make alternate claims to rights and entitlements. I argue that translating text and images into wearable data renders lesser-known technoscience stories visible and (more) knowable and transforms clothing (back) into material matters of public concern.

## Introduction: Getting into research

The skirt gathers up above my knees. I’m surprised, and relieved, that the sewn-in pulley system works. My office bears evidence of multi-dimensional iterations of this invention – paper models, small-scale fabric toiles [cheap material practice pieces, often in calico], post-it notes and white board sketches. But now I am in it. A second ago, it looked like an ordinary A-line floor length skirt. I pull waistband cords through stitched channels concealed inside the skirt’s centre front and rear seams. The inventor’s aim was to lift fabric up and out of the way of the bicycle wheels. It’s now a short cycling skirt. I release the cords. The fabric quickly falls to the ground. It’s back to a long walking skirt.

**Figure 1.**
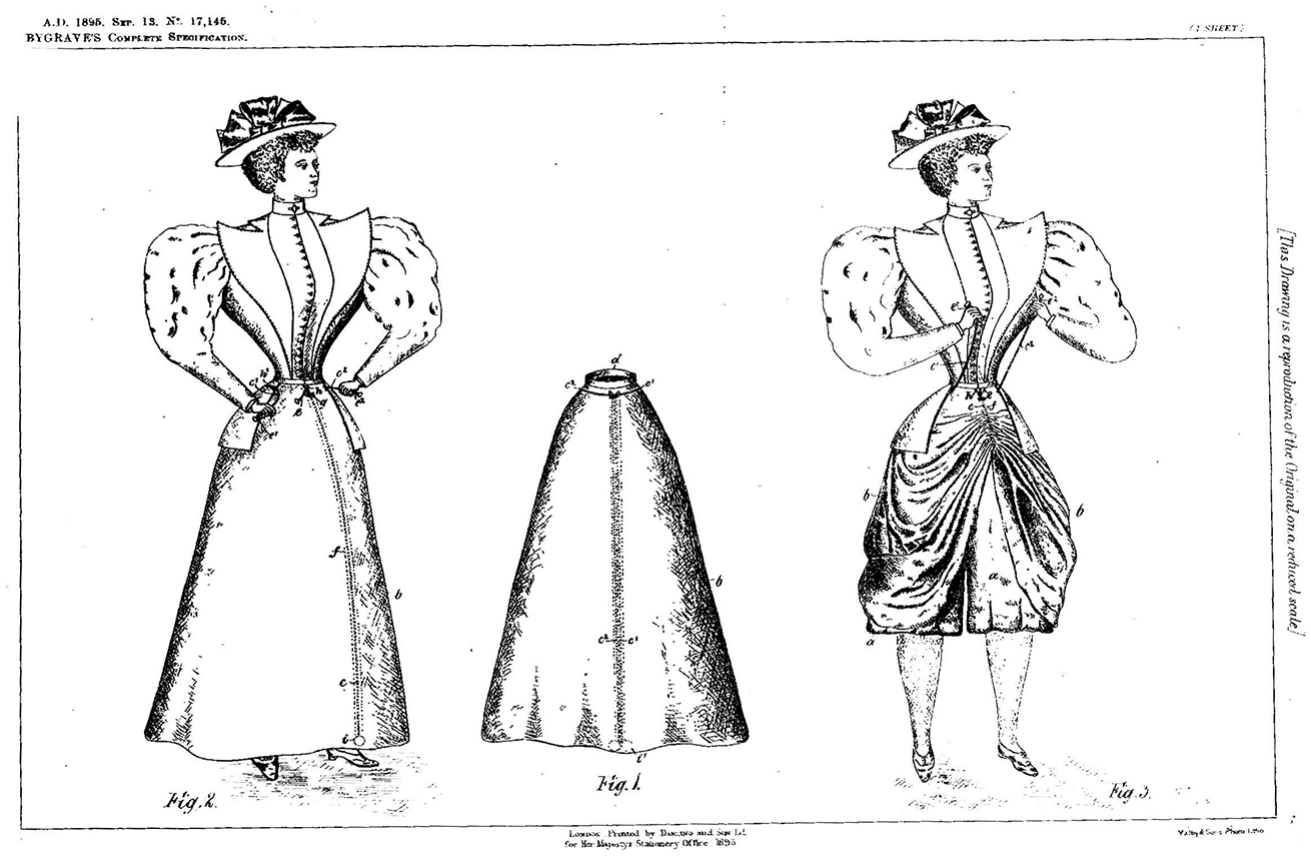
Drawings from Alice Bygrave’s (1895) convertible cycling skirt patent (Image used with permission of EPO.)

This ethnographic note comes from a messy sociology office where I am in the middle of reconstructing a convertible cycling skirt invented in 1895 by Londoner Alice Bygrave (Figure 1). A patent document and snippets in disparate archives were all that remained of her remarkable invention until this project. Bygrave grew up in a watch- and clock-making shop on the busy Kings Road in Chelsea. Her mother taught her to sew, her father delivered goods by bicycle and patented sprung saddles in his spare time, and her brother and sister-in-law were racing cyclists. Bygrave’s various skills and influences come together in this unique pulley-enabled walking and cycling skirt. I had been researching how inventors at the turn of last century used radical new forms of clothing to challenge socio-political restrictions on women’s bodies in public space and make alternate claims to citizenly rights and entitlements. A key question, in the scene above, was did this 127-year-old invention actually work? In this article I explore what *other* kinds of work and world-making the skirt did then and continues to do now, in its various multi-dimensional forms.

I start the discussion in the 1890s. Bygrave’s invention emerged during a unique intersection of socio-political, technical, and cultural change. A cycling craze swept the world at the turn of last century. Women were especially early adopters of this symbol of modernity, freedom, and independence. Historians estimate ‘there were at least several million women cyclists worldwide in 1896’, which ‘ranged from a third to half of all cyclists’ (Kinsey, 2011, p. 1122). While popular, cycling for women came with social and sartorial challenges. British newspapers reported terrible crashes causing disfigurement and even death when long skirts and petticoats caught in chainrings and pedals. Swapping long skirts for more ‘rational’ dress – the English Rational Dress movement campaigned against irrational fashions – such as bloomers, was safer. However, because cycling was deemed a ‘natural’ masculine pursuit, looking too much like a cyclist could be problematic. In addition to heightened social scrutiny, many suffered verbal and sometimes physical abuse from onlookers shocked by the behaviours of progressive ‘New Women’ seen to be carving out new gendered mobile practices.

Bygrave was one of many pioneering Victorian women who took what became known as the ‘dress problem’ into their own hands (Jungnickel, 2018a, 2021). They imagined, designed, made, and patented a vast range of inventive forms of clothing to enable early women cyclists to ride bicycles.^1^ People, and especially those denied power and influence in other areas, have long used clothing as a means of expression, to challenge conventions and renegotiate boundaries. At the turn of the last century, women lacked equal rights to men in relation to jobs, property, pay, and political representation. Cycling’s popularity encouraged many non-professional women, from women’s rights activists to ordinary dressmakers, to experiment with new ways of being in, moving through, and claiming public space. In this way, their use of clothing can be seen as sites of ‘non-verbal resistance’ (Crane, 2000, p. 99) and of ‘public struggle and contestation’ (Marres and Lezaun, 2011, p. 491).

Convertible cycling skirts generated multiple possibilities. Bygrave’s aim, according to her patent, was to ‘provide a skirt proper for wear when either on or off the machine’. Women didn’t just have to be cyclists or walkers. They could occupy multi-modal identities and positionalities. They could choose when and where to reveal their progressively attired and active bodies and cycle safely. Or they could conceal their intentions, when away from the bicycle, to minimize the risk of harassment. Basically, they could switch between these identities to dwell and move differently in public space. Not all inventions were as complex as Bygrave’s pulleys. Some used simpler mechanisms to adapt existing skirts into safe cycle wear. Regardless of the technology involved, ‘inventors searched for ways to easily transform the female cyclist, chameleon-like, back into her former self when dismounted’ (Helvenston Gray & Peteu, 2005, p. 31). Conversely, by forging new paths into legal and business worlds, the process of patenting offered public recognition for non-professionals. These various in/visible ‘acts’ and ‘performances’ helped inventors, and wearers of their inventions, carve out their own versions of civic rights and entitlements, otherwise unavailable to their sex (Hildebrandt, 2019; Isin, 2019).

Bygrave’s invention can be considered successful, by some criteria. Her skirt was patented four times (in Britain, Canada, United States and Switzerland), and commercialized and distributed by Jaegar, a British manufacturing firm (*The Lady Cyclist*, 1896, p. 1). The desire for a dual costume appealed to many. At one time, the ‘Bygrave “Convertible” Skirt for Cycling & Walking’ could be purchased across England and Scotland, in North America and even Australia. Despite this, accounts of female ingenuity in relation to clothing are not easily found in cycling or technology histories. Stories of inventive workarounds and material acts of resistance remain largely unremembered. This is partly because gendered technology practices have been vastly undervalued in the past (Star, 1999; Wajcman, 2013). Schwartz-Cowan (1983) reminds us that ‘the absence of a female perspective in the available histories of technology was a function of the historians who write them and not of the historical reality’ (p. 51). It also relates to a lack of surviving historic artefacts, an issue amplified with active wear, which is often worn out with use, and in this case deliberately designed not to be seen.

Haraway (2016, p. 12) argues that it ‘matters what matters we use to think other matters with’. Bygrave’s convertible skirt patent is lively even on paper, in age-spotted text and drawings. It conveys information about the inventor, including address and vocation, their perceived problem and proposed solution. The drawings offer multi-scaled views of the invention. This data is valuable, but how adequately can it communicate something worn intimately close to the body and purposefully hidden in plain sight? What can’t be summoned into the present with text and images alone? What other kinds of knowing does making make?

In the following, I build on a long history of reconstruction literatures in STS to describe and theorize the practice of researching, reconstructing, and reimagining text and images into wearable data. Along with material participation and citizenship theory, I argue that speculative sewing renders lesser-known technoscience stories visible and (more) knowable, and transforms clothing (back) into material matters and enactments of public concern (Hildebrandt, 2019; Isin, 2019; Marres, 2012). This involves being attentive ‘to the abilities of specific objects to disturb, provoke and suggest, and the critical role this plays in the making of political events’ (Marres, 2012, p. 140). I also speculate on what can be known about concealed inventions and secret activities of marginalized and under-represented groups. Sewing language fits well with this scholarly sartorial pursuit. I piece, layer, thread, unpick, patch, and stitch together theory, methods, practice, and archival materials. I trace inventors’ instructions provided in patents and discuss how practices of reconstruction, in tandem with close material encounters and speculative re-imaginings thicken the possibilities of data, understanding and knowing and in the process draw attention and raise questions about things hidden or taken for granted.

## Reconstruction and speculation in STS

Speculative sewing builds on a long history of reconstructions and replications in STS, history of technology, food sciences, textile studies, archaeology, and global Do-It-Yourself movements. Amongst others, researchers have cooked from archives (Connell & Nicosia, 2015), reconstructed a range of iconic electromagnetic and electrochemistry experiments (Cavicchi, 2006; Eggen et al., 2012), recreated textile arts (Bendall, 2019; Kuchera, 2018), reproduced Faraday discoveries (Höttecke, 2000; Tweney et al., 2005) and participated in hackathons and maker spaces (Irani, 2015). These make up some of what has come to be known as ‘making and doing’ projects (see Downey & Zuiderent-Jerak, 2021; Kenny et al., 2019). While diverse in practice and purpose, these projects recognize that not all data can be written, spoken or visualized. Some things must be experienced and known in other forms.

In STS projects, researchers emphasize how different kinds of experiments and hands-on material engagement can reveal complex socio-political critiques otherwise taken for granted or difficult to understand or access. Reconstruction affords a way into blackboxes: a means of seeing and understanding how relations of knowledge, power and politics are made, remade, and resisted that might otherwise remain invisible. Kenny et al. (2019), for example, write about students learning to build and use thermal flashlights who developed ‘nuanced critiques of the social relations inherent in an array of institutions and energy infrastructures that are otherwise taken for granted’ (p. 5). In the process, they learned to ‘see’ larger social, political and power issues built into infrastructures and devices, and were able to better grasp how perspectives, power, and gender relations become fixed in the making of technology. While this holds true for much STS, rather than taking something firm apart, reconstruction starts with parts and pieces them together. This means the process and happenings along the way are as much data as the artefact itself.

Critically, reconstructions are not about seeking to identify a distinct or single social reality. They are better thought about as speculative, multiple, and creative entanglements. Like research methods for Law and Urry (2011), reconstructions ‘are performative’ and ‘have effects; they make differences; they enact realities; and they can help to bring into being what they also discover’ (p. 393). As such, this article intersects reconstructions with speculative approaches in queer studies, technoscience, and design fabulation (Bryan-Wilson & Dunye, 2013; Forlano, 2019; Haraway, 2016; Maguire et al., 2021; Michael, 2016; Rosner, 2018). Like reconstruction, speculative methods invite researchers to ask questions about things inexistent, problematic or rendered invisible by familiarity. Michael (2016) explains how it is possible to ‘speculatively make “visible” the potentialities that emerge in everyday life’ and this enables researchers to ‘query the usual frames of reference through which we grasp the mundane’ (p. 655). Researchers can ‘experiment with what could be rather than what is or what should be’ (Forlano, 2019, p. 2816) and these ‘imaginative resources make way for living differently in the present’ (Rosner, 2018, p. 17). Approaches like these are especially important when things are damaged, fragmented or entirely missing in archives, as is the reality within many indigenous, queer or gendered histories. They can draw political attention to ‘telling blanks and perversely wilful holes’ (Bryan-Wilson & Dunye, 2013, p. 82). For some, ‘[i]dentifying those absences may be an important element of the historian’s task’ (Sheller, 2012, p. 3). Bryan-Wilson (2014, p. 83) writes about how ‘Dunye has consistently explored the affective potency that lies within historical records – and the gaps in those records – to explore how fictional archives might be necessary for queer lives in the present as well as for imagined futures’.

This kind of socio-technical intimacy is especially critical for clothing patents, because they describe artefacts designed to work with and on bodies. While this is true for all clothing, it is exaggerated for active wear like Bygrave’s invention. Less than half the story is revealed on the surface of a convertible costume viewed from a distance, on a hangar or folded in a box. And no doubt many a convertible skirt lies undetected, and unresearched, in personal wardrobes and museum collections for this reason. Entwistle (2015) argues that clothes are not lifeless ‘shells’. They hold traces of people who made, lived in and shaped them. Without bodies, clothes can only tell us so much. ‘When dress is pulled apart from the body/self, as it is in the costume museum, we grasp only a fragment, a partial snapshot of dress, and our understanding is thus limited’ as they ‘cannot tell us is how the garment was worn, how the garment moved when on a body, what it sounded like when it moved and how it felt to the wearer’ (p. 10).

This article also draws on material participation and expanded citizenship studies that challenge the top-down orthodox view of citizenship solely as an individual’s relationship to the nation-state citizenship (Hildebrandt, 2019; Isin, 2019; Marres, 2012). Instead, interdisciplinary scholars argue that citizenship is also enacted, performed and negotiated on many scales, including sensory, material and embodied mundane daily practice. Isin and Neilson (2008, p. 2) explain ‘acts of citizenship’ as a shift ‘from the institution of citizenship’ to ‘collective or individual deeds that rupture social-historical patterns’. Critically, these acts do not have to be radically interventionist (though they can turn out this way). Rather, they can be modest and mundane or, even as in the case of convertible skirts, deliberately concealed from view.

I also suggest that the concealed character of convertible skirts like Bygrave’s can be speculatively explored in terms of steganography. Kuchera (2018) explores steganographic practices, or ‘hiding information in plain sight’, in classic literature, patterns, anecdotes and fictional accounts to speculate on how women and other marginalized people have used a range of mundane practices like knitting, crochet, embroidery and quilting to share and record information and encode secrets. She reconstructs some of these examples, otherwise unavailable for study, to ‘speculate about the potential historical, present, and future use of such methods as a means of communicating and protecting information intended to be shared between members of marginalised or even openly persecuted groups’ (2018). How to get beyond the surface and into alternate citizenship stories concealed in clothing inventions is central to the next part of the article.

## Clothing patent data and methods

This article emerges from research in two related projects: the Economic Social Research Council funded *Bikes & Bloomers* (B&B) into British clothing patents from 1890 to1900 and the European Research Council funded *Politics of Patents* (POP) on global clothing patents (covering 94 countries) from 1820 to 2020.^2^ Both draw data from the European Patent Office, which holds over 120 million publicly available worldwide patents (see European Patent Office, 2022). This research explores how clothing inventors have over time attempted via mundane and ordinary means to resist, subvert or disrupt hegemonic norms. Rather than asking ‘who is the citizen?’ the question becomes ‘what makes the citizen?’ (Isin, 2009, p. 383).

While speculative sewing is applied across both projects, I discuss one case in detail. In B&B, I focused on early British cycling cultures to examine how women made creative claims for freedom of movement and civic participation via clothing. I identified eighty-six inventions for new models for or improvements to women’s cycling skirts, thirty-two of which were for convertible costumes patented in Britain at the turn of last century. Nearly half are by women. This period was the height of the cycling craze and patenting boom in the country. As discussed above, the ‘dress problem’ tempted many non-professionals, notably women, to patent ideas for the first time.^3^

Bygrave’s convertible skirt patent is a particularly useful example that I have been researching for the past eight years, with multi-layered insights generated from archives, reconstructions, ethnographic experiences in costume and public engagement activities as well as data generated from open access sewing patterns.^4^ As such, data takes many forms. In the following, I analyse patent text and drawings, ethnographic notes and photographs from the reconstruction process and inviting others into the research along with related newspaper, periodical and genealogical archives. Grounded theory was used across this and the larger corpus to code data to identify emerging patterns and themes (Charmaz, 2014).

Patents may be more familiar data in legal and business contexts than in social sciences, yet they have been shown to be valuable for technoscience study (Cochoy, 2021; Khan, 1996). Further to providing information as outlined above about the inventor, their identified problem and solution, they also hold voices of those otherwise silenced or erased in historic records. As Kenny et al. (2019, p. 11), argue, ‘the politics of visibility is not only about what data is made visible, but who is made visible through technology creation and data collection’. Patent archives hold a valuable record of women’s technoscientific practices unavailable elsewhere. Khan (1996, pp. 365–366) notes that historic patents ‘present a valuable perspective on female inventive activity and market participation in an era when marriage meant the virtual ‘invisibility’ of married women in terms of objective data’.

The research is also sensitive to who isn’t in the archive. We recognize that patent archives do not record all inventive ideas and what they do hold are significantly shaped by colonial legacies, gendered biases and class privilege. Despite this, they can still be useful starting points for alterative investigations into lesser-known, marginalised and expansive global socio-technical accounts (Foster, 2017; Khan, 2000). We also use them to ask different and larger questions. With a feminist decolonial technoscience approach, Foster explores how ‘gendered assumptions, variegated colonial histories, and materiality of patented objects’ can ‘make us think differently about struggles over knowledge and belonging’ (p. 7).

On the surface, patents appear to be classic black-boxed legal texts of power and politics. However, as much as the inventor may have liked, there is no single way to read and interpret this data. Even seemingly straightforward instructions do not preclude creative interpretation, workarounds, mess, and mistakes. Kuchera (2018) argues that it is worth remembering how ‘history has provided us with profound examples of reasons women, nonbinary people, and others once referred to as “gender minorities” may have wanted or even needed to encode messages – to hide information, or even themselves, in plain sight’. Patents may in fact harbour more secrets than they suggest on the surface and hold answers to questions we may not even think to ask.

## Analysis – in the office, on the stage and on the bike

The following sections explore insights generated from researching, reconstructing and reimagining Bygrave’s convertible skirt in different contexts. The first centres on an academic office in the process of translating text into clothing. The second, a public performance, draws attention to a breakdown in the pulley system and the ongoing care and repair required to maintain the invention. The third reflects on what happens when the invention leaves the research project and takes on (even) more meanings. Throughout, I highlight ‘an appreciation for mundane, everyday ‘low-tech’ artefacts and their ability to generate or firm up novel forms of citizenship’ (Marres & Lezaun, 2011, pp. 491–492).

### The extra-ordinariness of an ordinary technology

There are weights in the hem. I try to work out what kinds of weights, but the inventor isn’t very forthcoming. She just says it is a ‘concealed weight’ located at ‘the bottom edge of the skirt where the end of the cord is made fast to it’ and ‘should be a little heavier than the front of the skirt in order that the latter may be pulled down quickly as soon as the cord is released’. I realize that Bygrave resists being specific, as the weight needs to offset the combined weight of the cords, stitched channel and volume of skirt. Everything is interconnected.

Bygrave’s skirt has weights hidden in the front and rear hems. Two small but hefty masses are sewn into pockets concealed in folds of fabric that skim the floor. Insights can sometimes directly relate to time invested in a task. This is one such example. Bygrave’s weighted hems were overlooked in readings and omitted in initial iterations. In paper models and small-scale fabric toiles, I initially considered the fabric to be the ‘weight’ to be lifted. The additional weights did not seem relevant to the overall system. However, as I learned, the extra weights did other work and a deeper understanding about the extra-ordinary nature of this mundane part emerged in the making.

First, the weights highlighted invisible behind-the-scenes work. Many hours were spent researching what to use as weights, where to put them and how to secure them in place. I sourced curtain weights, circular in shape, and with a handy hole in the centre. Following Bygrave’s suggestion, I ‘made fast’ with the cords by knotting them to the weight before hiding them in the hems. I was the only one to see them and have only glimpsed them since during repairs. Apart from feeling them bang lightly against your ankles as you walk, they are otherwise hidden. I reflected on how this small yet time-consuming task was indicative of the invisible work undertaken by early women cyclists to ride bicycles in public spaces.

Second, the weights drew attention to the speed of the conversion. I had been researching the abuse early cyclists suffered for daring to cycle, let alone doing so in cycling wear. Rare first-hand accounts like this give a sense of a daily experience for some brave women: ‘Hooting and screeching were the usual accompaniments to every ride. Caps, stones, road refuse – anything was then flung at the hapless woman who dared to reveal the secret that she had two legs’ (Marshall, 1899, p. 40). Being identifiable as a cyclist on foot would have exposed the woman to potentially more harassment. The capacity to quickly switch from short cycling skirt to a conventional long walking skirt would have been highly valued. And Bygrave’s weighted hems did exactly that. They afforded a rapid response. When the cords are released, the skirt falls quickly to the ground, screening the legs and actions of the wearer. This was a key selling point of ‘The Bygrave “Quick Change” Cycling Skirt’, which was advertised as being ‘instantaneously raised or dropped’ (*Lady Cyclist*, 1896, p. 1).

Technofeminists have written at length about the importance of small, mundane and boring things, drawing attention to things easily overlooked and under-appreciated. Large-scale sociotechnical systems can appear very different to those who are actively restricted by them. It is then that ‘[o]ne person’s infrastructure is another’s topic, or difficulty’ (Star, 1999, p. 380). In this case, cycling offered freedom and independence to part of the population – a specific type of male citizen. It was easier for those whose bodies, socio-political freedoms and clothing ‘fitted’ with the bicycle, than it was for others. Inventions like Bygrave’s utilized available at-hand materials to adapt women’s bodies and clothing to fit, and quickly. Here, the very ordinariness of weights in the pulley system become extra-ordinary in their application.

**Figure 2.**
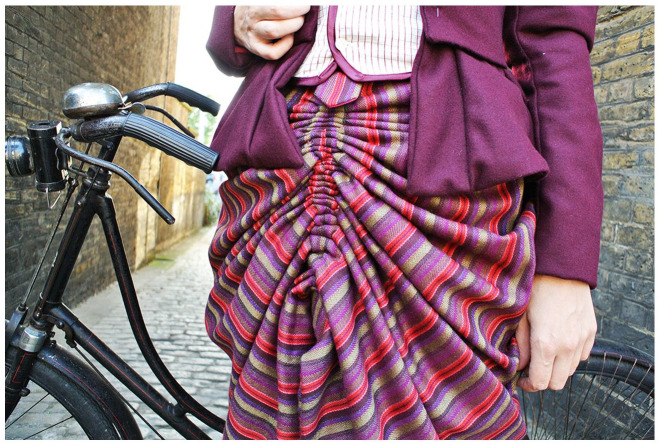
Using patent instructions, materials and the researcher’s body to understand the pulley system concealed inside Bygrave’s convertible cycling skirt (Author image).

Other related instances of the extra-ordinariness of the skirt emerged in relation to language. Bygrave advises readers that her converted cycling skirt should ‘festoon’ over the hips. This had the research team initially perplexed. Is this important to the design? Will we know it when we see it? It wasn’t until I made and put the costume on and tried it out on a bicycle that the festooning effect made sense. The pulleys gathered the skirt up at the centre front and back, enabling safe cycling, while draping fabric over the hips, which in turn concealed the side view of the legs while pedalling. This style reflected fashionable styles of the time, thus making the conversion more acceptable on the surface, even though underneath it enabled a radical act. I came to understand the practical, political, and aesthetic purpose of festooning and how the inventor, and wearers, used it to work-around barriers to their freedom of movement (Figure 2).

The value of these small examples lies in how they render visible larger socio- political issues and worlds. The complexity of the weights and the festooning effect were not evident from the initial reading of the text. It is unlikely that either would have generated attention in a journal article had I not reconstructed the skirt and dealt with both in practice. This is especially relevant in the context of archival records, which can seem simple, smooth, and distant (an issue made even more relevant in digital form). Reconstruction practices also provide useful reminders that innovation is not neat and orderly. Although I was following the step-by-step instructions provided by the inventor, the process was far from straightforward. It went side-ways, paused, and overlapped. This is a valuable finding, as others have discovered in practice. As Kenny et al. (2019, p. 22) write about student projects: ‘The scientific and technological method proved to be a hot mess rather than the step-by-step process represented in their textbooks.’ A more ‘common aspect of science in practice’, argue Eggen et al. (2012), is for it to be ‘untidy and unpredictable’ and this goes vastly ‘under-reported’ (p. 179).

The specificities of seemingly ordinary parts, unfamiliar language and processes takes time and concentration. All of which can attune the researcher to the research. ‘Taking part in historical experiments,’ Cavicchi (2006, p. 741) explains, ‘extended what the learners were able to do with their hands and with the materials, as well as what was there to observe and consider.’ This is not uncommon. In the process of making and using thermal flashlights, Kenny et al. (2019) note how the students became technoscientists. Bendall (2019, p. 382) writes similarly about her personal transformation after reconstructing historic French undergarments: ‘Primarily, this experiment taught me to think more like an artisan and less like a scholar, often the result of learning from mistakes.’ For me, speculative sewing slowed down the research, thickened and enriched the findings, expanded my sewing skills and sharpened my ability to more closely read, interpret, and question texts and objects.

### More-than-a-skirt: Failure, repair and maintenance

A cord snaps. Only one side of the skirt converts. Half the fabric is gathered up to the waist. The other half hangs closer to the floor, held down by the weights in the hem. I am standing in the skirt, on a stage, in the middle of a talk and demonstration to a public audience. I reach down to manually lift it up and ‘festoon’ it over my hips to show how it works, while explaining how I have just broken it.

This might seem like a terrible thing to happen in a public event. Like a PowerPoint slide show not working, a malfunctioning costume adds a stressful element to a presentation. The aim of a convertible skirt was to afford dual activities: for women to walk and cycle. To do this, it had to pass as an ordinary floor-length walking skirt and operate effectively as a short cycling skirt with loose fabric gathered up safely out of the way of the moving wheels. To protect the wearer against potential harassment, it had to transition quickly and smoothly between positions. By breaking the system, I got stuck in between, in a liminal space in which neither worked. I had to demonstrate what should have happened while also explaining what had gone wrong. But it also gave me an opportunity to segue into a discussion about it as more than a skirt, in its need for ongoing care and repair. In this context, the skirt continued to work. Like the classic Bush Pump ‘B’, ‘whether or not its activities are successful is not a binary matter’ (De Laet & Mol, 2000, p. 252).

Patented inventions in the form of convertible cycling wear are difficult to understand in texts and static form. As indicated above with the weights, convertible skirts need mobile bodies to make sense. Due to their peripatetic nature, they are activated by arms, hips and legs and the moving machinery of the bicycle. They rarely work away from bodies. Their stories are enacted and enlivened through dynamic networks of humans and things. This, in turn, makes them vulnerable. In the case above, I failed to make the cords strong enough to withstand the stress in the pulley system. Although I could find no evidence of this in the archives, with regular use we can assume wearers at the time would have dealt with similar issues. This reinforced awareness that this was more than just an ordinary skirt. I came to understand how wearers would have had to care, repair and maintain it differently than other items in their wardrobes.

Interruption and breakdown, as many in STS have argued, can reveal the complexity of the systems at work, as well as points of repair and maintenance. Star (1999, p. 382) writes: ‘The normally invisible quality of working infrastructure becomes visible when it breaks.’ And we can appreciate from the Bush Pump ‘B’ the strength and flexibility of a socio-technical artefact even when it’s not doing what we might expect. Connell and Nicosia (2015) write about accidentally burning a batch of candies while cooking from the archive. They reflect how ‘[o]n the one hand, this mistake prevented us from tasting the recipe’s intended flavor; on the other hand, it’s likely that someone preparing these candies on an open hearth 300 years ago might have burned them slightly, too.’ Labouring over various iterations of the pulley system in Bygrave’s skirt, repairing and caring for it, I imagined how she too might have tinkered late into the night on the design-in-progress, pulling it on and off her body, dressing others and trying it out on bicycle rides.

Another example of failure is notable for similar reasons. A few years ago, I commissioned a dressmaker to reproduce another version of Bygrave’s skirt for an exhibition. I was short of time, had a budget for the project and assumed the finished skirt would be much more professional and hard-wearing. I took delivery the night before the show. I expected it to look great. It did. I was surprised, though, that it did not work. The pulley system failed to convert the walking skirt into a cycling skirt. It got stuck halfway. I had to unpick and remake it from the inside out. Although initially frustrating, I came to understand that the dressmaker failed to see it as a dual artefact with equal subjectivities. Making Bygrave’s cycling invention required a commitment to thinking about it as a technoscientific wearable, not just as a skirt.

While I try to avoid costume failures, these examples are useful experiences. Reconstructions in STS rarely aim for perfect or static outcomes. Things change, and as they do, they reveal new things. The process sometimes doesn’t even get close to a finished artefact. Reconstruction offers a way to go beyond the surface and into the context, process or object itself and beyond, to speculate on how wearers dealt with certain situations, such as the making, ongoing care and maintenance of skirts as technological artifacts. Appreciation and insights deepen when seemingly simple processes turn out to be much more complicated than expected. ‘Anomalies or unexpected behaviours,’ writes Cavicchi (2008, p. 419), ‘attract [the] historical experimenter’s interest, giving rise to new investigations and observations that branch out by nonlinear paths.’

I would not have understood much of this without the experience of continued use. Bygrave’s skirt has been broken and repaired many times. Weights have come loose, cords replaced, holes patched, buttonholes mended, and buttons restitched. Rather than hide repairs, I take reference from the visible mending movement to fix the issue, while layering the experience and thickening the data. As Durrani (2021, p. 796) writes about one of her ethnographic informants: ‘Caroline’s mending, like her cardigan, has continuously informed her body, reformed her garment, and refined her practice.’ This thickening of data practically and theoretically fills gaps rendered visible in the reconstruction process. Tweney et al. (2005, p. 139) argue similarly about the value of extra data: ‘The apparent gaps between Faraday’s experiments and his finished (and unfinished) mental models must be filled with additional information to understand his work.’ Even 125 years after it was invented, the Bygrave skirt continues to tell new stories, take on new shapes and spaces in public.

### Material dialogues in public

Two audience members arrive in costume to one of my public talks. They had made use of the open access patterns provided on the project website to construct their own versions. Unexpectedly, they are wearing a combination of patented pieces; bloomers from one inventor, skirt from another, cape from a third. I was accustomed to thinking about patents as unique sets, not as interchangeable assemblages. Yet, this is quite possibly what wearers of the time would have done.

Nine sewing patterns inspired by the initial patent research are available on the project website. There are five convertible skirts, including Bygrave’s, two pairs of bloomers, a jacket and an all-in-one undergarment. These free open-access downloadable pattern packs have been downloaded 35,000 times, to date. Like many who send me images and share results on social media, the women at the event above had taken up the challenge to make and wear their own versions of historic cycle costumes. They also told their own stories about them. They were friends and had helped each other sew, sharing skills, sewing machines, patterns and fabric. They cycled together to the event. They paired period pieces with modern ones and made these historic inventions their own, merging patents and timelines as easily as they stitched materials. These varied engagements and articulations opened new human and material and past and present conversations. I have emphasized throughout the project that just as there was no one way to respond to the challenges facing early women cyclists, there are many ways to interpret these patents and our patterns. I welcome responses, questions and feedback from those downloading and making their own.

Of course, one of the primary reasons to reconstruct Bygrave’s skirt is to see if and how it worked. I had established that I could walk, and it converted smoothly between modal forms. But how did it feel on the bicycle? Did it live up to inventor’s aim to work ‘on or off the machine’? The women in costume above attested to the success of the invention by cycling to the event. I also found it worked in practice. The cords gathered the fabric effectively up and out of the way of the moving wheels and the festooning effect over the hips covered the sides of my legs, creating an attractive draping effect. It is not uncomfortable to sit on; in fact, the folds of fabric cushioned the seat. And as indicated above, the “Quick Change” conversion lived up to its name. Provided the skirt was made of a non-creasing fabric, there was no residual evidence of use.^5^

Proximity is often a central concern in reconstruction and speculation projects. Many claim that distance is reduced, and the imagination sparked when you can see, feel, touch or even taste the lives of others. Höttecke (2000) argues that research is limited if ‘restricted to textual sources’ or just ‘intellectual work’ – though of course work in archives can involve relevant sensory experiences – and is instead better thought about as a ‘vivid experience’ which ‘comprises the difficulties of experimenting, the development of experimental skills as well as the possibility of sensuous experiences’ (pp. 343–344). This is especially critical in cases like Bygrave’s skirt, where there are things that cannot be seen and need to be experienced in other ways. Connell and Nicosia (2015) explain something similar with respect to their cooking project: ‘What this project has allowed us to do is incorporate other senses into the research process: smell and touch, but taste, too. … In this way, the archives become much closer to our daily lives and the lives of our readers.’

An audience member at a public event highlighted yet another advantage of the costume. Roads were largely unsealed at the time of invention. They were dusty and often muddy, which made cycling a potentially dirty activity. For women it was imperative to avoid being seen or arriving at a destination dirty, because, as Tickner (1987, p. 37) notes: ‘Feminists were regularly caricatured as over- or under-sexed, ugly, hysterical, masculine or incompetent.’ Convertible costumes, like Bygrave’s, ensured that even if the cyclists’ legs and undergarments became splashed or soiled, the skirt remained largely protected in its raised position. When released, it completely covered the wearer’s legs and recent activities. This revealed yet another concealed feature of the design. Skirts like Bygrave’s were deliberately designed to do more than might be anticipated from the surface. Kuchera (2018) writes that ‘[t]he potential of crafts often dismissed as “women’s work” as a means to hide information in plain sight is tantalizing’. It also turns out to be a timeless issue. The cyclist telling the story demonstrated by pointing to their own mud splattered legs and, in the act, linked the past to the present.

Unlike immutable mobiles, convertible costumes are never static. They make different kinds of sense on varied bodies and in situated practice. They can be assembled in unexpected ways. They entice people into the research, by disguising academic research as an appealing piece of clothing. They create new publics, elicit surprising invites and travel on bodies to unexpected places. De Laet and Mol (2000) explain how Morgan, the inventor of the Bush Pump ‘B’, visits devices in situ not to keep them ‘intact, shining like new’, but rather ‘tries to learn from the way the pumps have evolved on site, from the ways in which users have repaired and adapted their devices’ (p. 251). Much as there is no single way to follow the inventor’s instructions, there is no single way to make, wear, repair or perhaps ever fully know convertible skirts. Like Morgan, I also like being surprised by the constant inventiveness the inventions incite.

## Conclusion: Speculatively sewing three dimensional arguments

This article concerns the practices of researching, reconstructing and reimagining archival clothing patent data, focusing on convertible cycling skirts at the turn of last century in the Global North. I explore how inventors used new forms of clothing to creatively workaround restrictions to their freedom of movement. These socio-material acts and enactments of citizenship took various forms. The concealed nature of the skirts enabled wears to inhabit informal multi-modal identities, while the patenting process enabled inventors to claim their ideas in official contexts. As a result, convertible skirts offered inventors and wearers visible and less visible ways of participating in socio-political life and claiming new ways of being in and moving through public space.

Reconstructions can be especially critical when stories are missing, flattened or secret. Data on early inventors, especially women and marginalized groups, is fragmented, sparse and difficult to piece together. Convertible cycle wear exaggerates this issue as inventors deliberately designed costumes not to be seen. Reflecting on steganographic practices, I investigate what lies beneath the surface of standard text and image-based data. I discuss insights emerging from taking inventions out of the archive (and university), making them material and putting them onto people’s bodies in varied contexts. These experiences reveal some of the secrets hidden in the skirts and pointed to the possibility of more. I highlight clothing’s role as multi-dimensional arguments that can be interrogated, inhabited and shared in different ways and how it transforms clothing (back) into material matters of public concern. Throughout, I describe and theorize Bygrave’s skirt in relation to the range of work it did then for early cyclists, and how it continues to hold relevancy for contemporary audiences.

Speculative sewing uses reconstructions of the past, to better understand the present and imagine different futures. It’s a means to think with and through the elements that make up sociotechnical worlds, of ‘making things to make sense of things’ (Jungnickel, 2018b). Some research projects benefit from being translated, made messy, material, or three-dimensionalized. Amongst other things, it invites contemporary audiences to speculate about their own lives, thickens data with fresh and relevant connections and encourages socio-political dialogues between century old experiences and today’s issues.

Finally, what emerges in both practice and subject analysed above is action. These late-nineteenth century women inventors didn’t just accept or challenge constraints, they acted on them. They questioned, re-imagined and made alternate ways of being in the world, put their designs on bodies, tested them in public space and then by patenting, secured their future in public record. By intimately tailoring the surface of bodies with radical forms of clothing they created new landscapes upon which to challenge conventions, change behaviours and expand possibilities of active mobile citizens. Speculative sewing holds the potential of something similar by transforming academic work into multi-dimensional and sensory forms that move us and others in new ways.

## Notes

1. See Jungnickel (2018a) for full list of British women’s cycle wear patents from 1890-1899.

2. See www.PoliticsOfPatents.org and http://BikesandBloomers.com/.

3. For more discussion about women patentees in this period, see Helvenston Gray and Peteu (2005), Khan (1996, 2000) and Jungnickel (2018a, 2021).

4. See http://bikesandbloomers.com/patterns/ to access the free sewing pattern packs

5. See http://BikesandBloomers.com/ for video of this costume in action
